# The Link between Knowledge, Attitudes and Practices in Relation to Atmospheric Haze Pollution in Peninsular Malaysia

**DOI:** 10.1371/journal.pone.0143655

**Published:** 2015-12-08

**Authors:** Laura De Pretto, Stephen Acreman, Matthew J. Ashfold, Suresh K. Mohankumar, Ahimsa Campos-Arceiz

**Affiliations:** 1 Mindset: Interdisciplinary Centre for Tropical Environmental Studies, The University of Nottingham Malaysia Campus, Jalan Broga, Semenyih, Selangor, Malaysia; 2 Department of Applied Psychology, The University of Nottingham Malaysia Campus, Jalan Broga, Semenyih, Selangor, Malaysia; 3 School of Politics, History & International Relations, The University of Nottingham Malaysia Campus, Jalan Broga, Semenyih, Selangor, Malaysia; 4 School of Biosciences, The University of Nottingham Malaysia Campus, Jalan Broga, Semenyih, Selangor, Malaysia; 5 Department of Biomedical Sciences, The University of Nottingham Malaysia Campus, Jalan Broga, Semenyih, Selangor, Malaysia; 6 School of Geography, The University of Nottingham Malaysia Campus, Jalan Broga, Semenyih, Selangor, Malaysia; University of Maryland at College Park, UNITED STATES

## Abstract

Transboundary haze episodes caused by seasonal forest fires have become a recurrent phenomenon in Southeast Asia, with serious environmental, economic, and public health implications. Here we present a cross-sectional survey conducted among people in Kuala Lumpur and surrounds to assess the links between knowledge, attitudes, and practices in relation to the transboundary haze episodes. Of 305 respondents, 125 were amateur athletes participating in a duathlon event and the remainder were surveyed in an inner-city shopping mall. Across the whole sample, people who possessed more factual information about the haze phenomenon showed significantly higher levels of concern. Duathletes were more knowledgeable than non-duathletes and also more concerned about the negative effects of haze, especially on health. For all people who regularly practice outdoor sports (including people interviewed at the shopping mall), higher levels of knowledge and concerned attitudes translated into a greater likelihood of engaging in protective practices, such as cancelling their outdoor training sessions, while those with greater knowledge were more likely to check the relevant air pollution index on a daily basis. Our results indicate that the provision of accurate and timely information about air quality to residents will translate into beneficial practices, at least among particularly exposed individuals, such as amateur athletes who regularly practice outdoor sports.

## Introduction

Air pollution is a major threat to human health and life satisfaction, and the haze phenomenon in Southeast Asia represents a singular case due to its primary origins in land-clearing for oil palm plantations and other agricultural purposes, and the political implications of its transboundary nature. Haze has been defined as “[a] weather phenomenon that leads to an atmospheric visibility of less than 10 km due to the amount of suspended solid or liquid particles, smoke and vapor in the atmosphere” [1]. Haze spreading mostly from forest fires and burning of tropical peatland in Indonesia affects Malaysia and Singapore (thus ‘transboundary’) on a seasonal basis, with greater severity during drought conditions [2]. While the most severe haze episode occurred in the 1997–98 season, which created a major environmental crisis affecting some 70 million people [3], dangerous and unsightly haze events have been a recurrent part of the lives of Malaysian residents for more than thirty years now. Putting aside the environmental damage caused by widespread forest burning and smoke entering the atmosphere, the haze has immediate effects on human beings via damage to tourism, transport, urban and rural aesthetics, food and water quality, and interruption to agriculture and other productive activities [3].

The everyday lifestyle of Malaysians is negatively affected by haze, with media reports having presented it as disruptive of sporting and other outdoor events [4]. High levels of air pollution have been associated with a reduction in life satisfaction [5], and a recent study into the human impact of the 2013 haze event in Singapore indicates that some degree of psychological stress is suffered by those experiencing a haze crisis [6]. Perhaps of greatest concern, however, is the negative impact of air pollution on human health. In regards to the haze phenomenon in particular, the increase in airborne fine particulate matter during haze periods has been shown to produce adverse acute health effects [7,8]. The long-term health impact of air pollution has been recently indicated by a quasi-experimental Chinese study, which found increased cardiorespiratory mortality, and a corresponding decrease in life expectancy, amongst populations exposed to air pollution [8]. It can therefore be anticipated that exposure to haze amongst residents of peninsular Malaysia will also have chronic health implications.

With a political solution to the haze crisis remaining elusive, studies of human perception and behaviour in response to the phenomenon are urgently needed. The public perception of urban air pollution has elsewhere been indicated as a key driver of personal behavioural change [9]. Most clearly explained in the research guidelines of Medicins du monde [10], qualitative studies based on a Knowledge-Attitudes-Practices (KAP) model are a common method for understanding and analysing human responses to particular phenomena, especially in the field of health studies [11,12]. The connection between people's attitudes and practices is well established in psychology, explained through the Theory of Planned Behaviour [13]. The role of various antecedents, mediators and moderators in the relationship between attitude and practices has been investigated, with special importance given to knowledge in relation to environment- and health-related attitudes and behaviour. Links between knowledge, attitude and practices have been derived internationally in relation to health issues [14], and there is support for a knowledge-practices link among Malaysians [15]. For specifically environmental matters, the Ecological Attitude-Knowledge Scale [16] is one well-known effort to determine the knowledge-attitudes link, and a comprehensive meta-analysis has found knowledge and perceived threats to personal health to be amongst the strongest factors affecting environment-related practices [17]. Factors such as education level and income have been shown to have an effect on people’s environmental- and health-related knowledge and practices [18]. Education has also been shown to be a significant factor in the knowledge of environmental problems amongst an Indonesian sample [19]. Moreover, health conditions related to air pollution have been shown to increase the levels of knowledge of air quality and how it relates to respiratory diseases [18].

Here we use a KAP approach to test whether there is a positive link between people’s knowledge, attitude, and adoption of protective practices in relation to atmospheric haze and whether these (KAP) are affected by the level of risk exposure of individuals. Our high-risk population were amateur athletes taking part in a duathlon competition at the end of the 2014 haze season in Peninsular Malaysia. General air pollution studies show that the haze represents greater health risks for people practicing outdoor sports regularly [20]. Duathlon is a sport that combines cycling and running and that requires intensive training, generally outdoors. We therefore assumed that amateur duathletes are a self-aware higher-risk population and that such vulnerability would affect their levels of awareness, concern, and protective behaviours to mitigate this risk. Specifically, we aimed at testing the following three hypotheses:

1. The higher the awareness (Knowledge) of the haze phenomenon, the higher the concern (Attitudes) about it that people will report;
2. The higher the awareness (Knowledge) of, and concern (Attitudes) over, the haze phenomenon, the more likely people will be to engage into protective actions (Practices) against it; and
3. The regular practice of outdoor sports mediates people’s KAP response to the haze, with amateur duathletes being more aware (Knowledge), more concerned (Attitudes) and engaging in more protective behaviours (Practice) than the general public.

We expected people who know more about the phenomenon, in terms of its origins, occurrence and frequency of severe episodes, instruments to check its severity, and economic and health-related consequences, will report more concerned attitudes towards it, compared to people with a lower level of awareness. We also expected people with greater knowledge and more concerned attitudes about the haze to consequently engage more in effective protective behaviours. People who regularly practice outdoor sport are more exposed to the health-related consequences of haze, as well as being more exposed to the visible dimension of the phenomenon. Thus, we expect them to show more awareness, and (consistent with hypotheses 1 and 2) also a higher degree of concern and practice of protective behaviours in relation to the haze. Data supporting these hypotheses will help to establish the importance of providing accurate and timely information regarding haze episodes to residents, especially those belonging to high risk groups such as outdoor sportspeople and people with respiratory health ailments.

## Materials and Methods

### Participants

We conducted a cross-sectional survey investigating knowledge, attitudes and practices in relation to transboundary haze. Two groups were selected and surveyed on the same weekend. One group were amateur duathletes taking part in a duathlon event and the other (control) group were members of the general public wandering through a popular shopping mall. In total, 305 respondents participated in the survey using this purposive sampling approach. By including the duathlon event, we ensured that the sample included both people who regularly practice outdoor sport and people who do not, thus allowing for comparison between sportspersons and others.

### Ethics statement

Ethics approval for this participant contact study was obtained from the Research Ethics Committee of the Faculty of Social Science, University of Nottingham Malaysia Campus. Research was conducted in line with the University of Nottingham Research Code of Conduct. Written consent was not required, and verbal agreement to participate in the survey was considered as consent. Participants were informed before commencement that they could withdraw at any stage while being surveyed, and that such withdrawal would render their entire participation void. They were also informed that data collection was anonymous, and that there would be no capacity for any individual to be identified based on their survey responses. The Ethics Committee gave formal approval of this approach to participant consent and data handling.

### Instrumentation

The survey instrument was created in two languages: English and Bahasa Melayu. Amongst countries in which English is not the primary language, Malaysia has the highest level of English competency in Southeast Asia, but there is nonetheless a significant minority of residents who are more comfortable with the national language [21]. The survey (S1 and S2 Files) was composed of four parts:

1. Background and demographic descriptors;
2. Haze knowledge questionnaire (composed of 10 true/false/no-answer questions, created by the investigators for the purpose of this study to test awareness of the haze and its implications in relation to health, international relations and economic costs);
3. Haze attitude questionnaire (composed of 17 items on a 5-point Likert scale, and 4 additional questions investigating the focus on haze concerns, created by the investigators for the purpose of this study to test attitudes towards the haze);
4. Wellbeing scale (adapted by the investigators from Diener’s standard wellbeing instrument [22]).

#### Procedure

Surveys were administered in two locations: at the duathlon event and at a shopping mall, in order to guarantee variability in the frequency and intensity of sport practice. The subsample at the duathlon event was entirely constituted by amateur athletes taking part in the race, not mere spectators or onlookers. The duathlon took place in Port Dickson, a popular coastal resort town approximately 95km from the centre of Kuala Lumpur. The shopping mall–Mid-Valley–is located in central Kuala Lumpur. The administration of the surveys took place over the same weekend (1^st^ and 2^nd^ of November, 2014) and the survey was conducted simultaneously at both locations in order to eliminate the possible influence of variability in atmospheric conditions. The selected dates were at the conclusion of the August-October peak haze season. In total, 125 surveys were administered at the sporting event (41% of the total respondents) and 180 surveys were administered at the mall (59%), providing a total sample size of 305.

Surveys were administered in pencil-and-paper format via an interview method. A group of 10 trained research assistants (RAs) administered the surveys to the participants in the two locations, supervised by three of the authors. Given the complexity of the four-part survey paper, administrators interviewed respondents and remained available to answer questions, rather than handing the surveys out for later collection. Surveys were administered either in English or in Bahasa Melayu, according to respondent preferences. Most RAs were bilingual, and they acted in pairs selected to ensure that there was always at least one RA with Bahasa Melayu fluency in each pair.

### Data treatment and analysis

Collected data was coded numerically to obfuscate identifying information on participants. With regards to the knowledge scale, participants received a score of 1 for each correct answer, a score of -0.2 for each incorrect answer and a score of 0 for each answer of "I don't know". A mildly negative score was given to distinguish those respondents who made an error from those who acknowledged that they did not know the answer. A mean ‘knowledge’ score was then determined for each participant. With regards to the attitudes scale, a mean ‘attitude’ score was calculated from the answers to each item on a 5-point Likert scale. Descriptive as well as inferential statistical analyses were subsequently performed; statistical notations can be found in Table 1.

**Table 1:** Statistical Notations.

t	t score
df	degrees of freedom
SD	standard deviation
SE	standard error
F	F ratio
B	the least squares estimate of β

#### Preliminary analyses

In order to test the construct validity of the haze attitude questionnaire, a principal axis factor analysis was conducted over its 17 items with an oblique (Direct Oblimin) rotation method. The Kaiser-Meyer-Olkin Measure of Sampling Adequacy for the factor analysis was meritorious (KMO = 0.81). An initial analysis was run to obtain eigenvalues for each factor, and small coefficients (absolute value < 0.4) were suppressed. Four factors had eigenvalues above the Kaiser criterion of 1.0, however the scree plot showed an inflection that would justify retaining just two factors. A reliability analysis was then conducted on these two factors, producing for the first factor, α = 0.813, and for the second factor, α = 0.119. Thus only the first factor was kept, leaving a single factor for the haze attitude questionnaire, composed of 10 items. The items clustering on this factor suggested that it represented concerned attitudes over the haze; thus the Attitudes element of the Knowledge-Attitudes-Practices method was labelled ‘concern’ and will be described as such hereafter.

Given that Diener’s wellbeing scale has not been widely used in previous research in Malaysia, and given that a shortened version of it was adopted for the current study, a reliability analysis was conducted for such scale as well, in order to check its reliability for the target population. That reliability was very low (α = 0.28), and thus the scale was considered unreliable for the sample and no further analysis was conducted that involved wellbeing scores. Wellbeing was an adjunct item inessential to the hypotheses and therefore the unreliability of this scale was not considered detrimental to the validity of the study as a whole.

The effect of all variables on knowledge and concern levels were first analysed in univariate analyses in the form of chi-squared tests of association, t-tests, or Pearson correlations, as required by the structure of the data. In the case of awareness and attitudes, we also ran analyses of covariance to test the effect of the different variables simultaneously. These analyses were run over two different datasets: (a) the whole dataset; and (b) a subset of respondents made up of Malaysian nationals who were interviewed at the shopping mall. The rationale for this second analysis is that Malaysian respondents at the shopping mall represent a more homogenous population, and thus some patterns might be easier to discern.

In order to remove non-significant variables, analyses of covariance were conducted in the R statistical environment [23] following Crawley [24]. First the full model was applied to the data, including all variables of interest, and then non-significant variables were removed manually one-by-one until only variables significant at the p < 0.05 level remained. The full model fitted onto the whole sample included the following variables: population (duathlon event vs. shopping mall), gender, education level (measured in four levels), whether the respondent has children, nationality (two levels: Malaysian vs. non-Malaysian), whether respondents suffered any relevant health condition, and whether they regularly practice outdoors sports. The concern model also included knowledge level as a covariate. The full model applied on the subsample of Malaysians at the shopping mall included the same variables with the exclusion of population and nationality (both of which were defined in the subsample selection).

## Results

### Sample demographics

The populations at the duathlon event and the shopping mall differed in a number of demographic parameters (Table 2)–at the shopping mall respondents were younger (26.1 ± 9.7 vs. 33.4 ± 8.6 years of age; *p*<0.05), more likely to be female (58.5% at the mall, 19.2% at the duathlon; *p*<0.05), less likely to have children (23.9% at the mall, 40.1% at the duathlon; *p*<0.05), had a lower income level, and a higher percentage of them reported health conditions that make them especially sensitive to poor air quality (32.8% at the mall, 12.0% at the duathlon; *p*<0.05). There was no difference in education level between the populations (*p*>0.05).

**Table 2:** Sample demographics and comparisons among respondents at the shopping mall and the duathlon participants.

		Shopping mall	Duathlon	*statistic*	*df*	*p*-value
**Number of surveys (N)**		180	125			
**Age (mean years ± SD)**		26.1 ± 9.7 (N = 175)	33.4 ± 8.6 (N = 125)	t = 6.89	1	3.7e-11
**Gender**				χ^2^ = 44.72	1	2.3e-11
	Male	58.4% (N = 178)	19.2% (N = 125)			
	Female	41.6%	80.8%			
**Nationality**				χ^2^ = 2.64	1	0.104
	Malaysian	86.0% (N = 178)	78.0% (N = 118)			
	Non-Malaysian	14.0%	22.0%			
**Ethnicity**				χ ^2^ = 17.07	3	6.85e-4
	Malay	47.1% (N = 153)	59.8% (N = 92)			
	Chinese	30.7%	35.9%			
	Indian	20.9%	2.2%			
	Others	1.3%	2.2%			
**Education**				χ ^2^ = 0.75	3	0.86
	Primary	1.1% (N = 176)	0.8% (N = 125)			
	Secondary	20.5%	16.8%			
	Tertiary (graduate)	64.2%	67.2%			
	Postgraduate (masters/PhD)	14.2%	15.2%			
**Income**				χ ^2^ = 14.92	4	0.005
	< RM2500	34.6% (N = 104)	17.8% (N = 90)			
	RM2500-5000	33.7%	26.7%			
	RM5000-7500	18.3%	22.2%			
	RM7500-10,000	10.6%	10.0%			
	> RM10,000	2.9%	23.3%			
**Have children**		23.9% (N = 180)	40.1% (N = 125)	χ ^2^ = 9.18	1	0.003
**Sensitive to poor air quality**		32.8% (N = 177)	12.0% (N = 125)	χ ^2^ = 16.13	1	5.9e-5
**Practice outdoor sports regularly**		57.0% (N = 179)	96.8% (N = 125)	χ ^2^ = 57.68	1	
	Hours/week* (mean ± SD)	6.1 ± 7.7 (N = 27)	9.4 ± 5.1 (N = 122)	t = 2.16	31.3	3.1e-140.039
**Check API/PSI daily**	General	4.7% (N = 129)	13.7% (N = 124)	χ ^2^ = 5.22	1	0.022
	Before practicing outdoor sports*	14.5% (N = 76)	40.5% (N = 121)	χ ^2^ = 13.72	1	0.0

N values under parentheses in Shopping Mall and Duathlon columns refer to the effective number of valid answers in that group (i.e. in some cases people opted not to answer certain questions).

* these questions were asked only to people who practice outdoor sports regularly.

As intended by the research design, the two populations differed markedly in their outdoor sporting habits; respondents at the shopping mall were much less likely to practice outdoor sports on a regular basis (57.0% at the mall, 97.0% at the duathlon; *p*<0.05). Among those who state that they practice outdoor sports regularly, respondents at the shopping mall do so for shorter periods (6.1 hrs/week at the mall, 9.4 hrs/week at the duathlon; *p*<0.05; Table 2).

### Type of haze impacts

**Fig 1:**
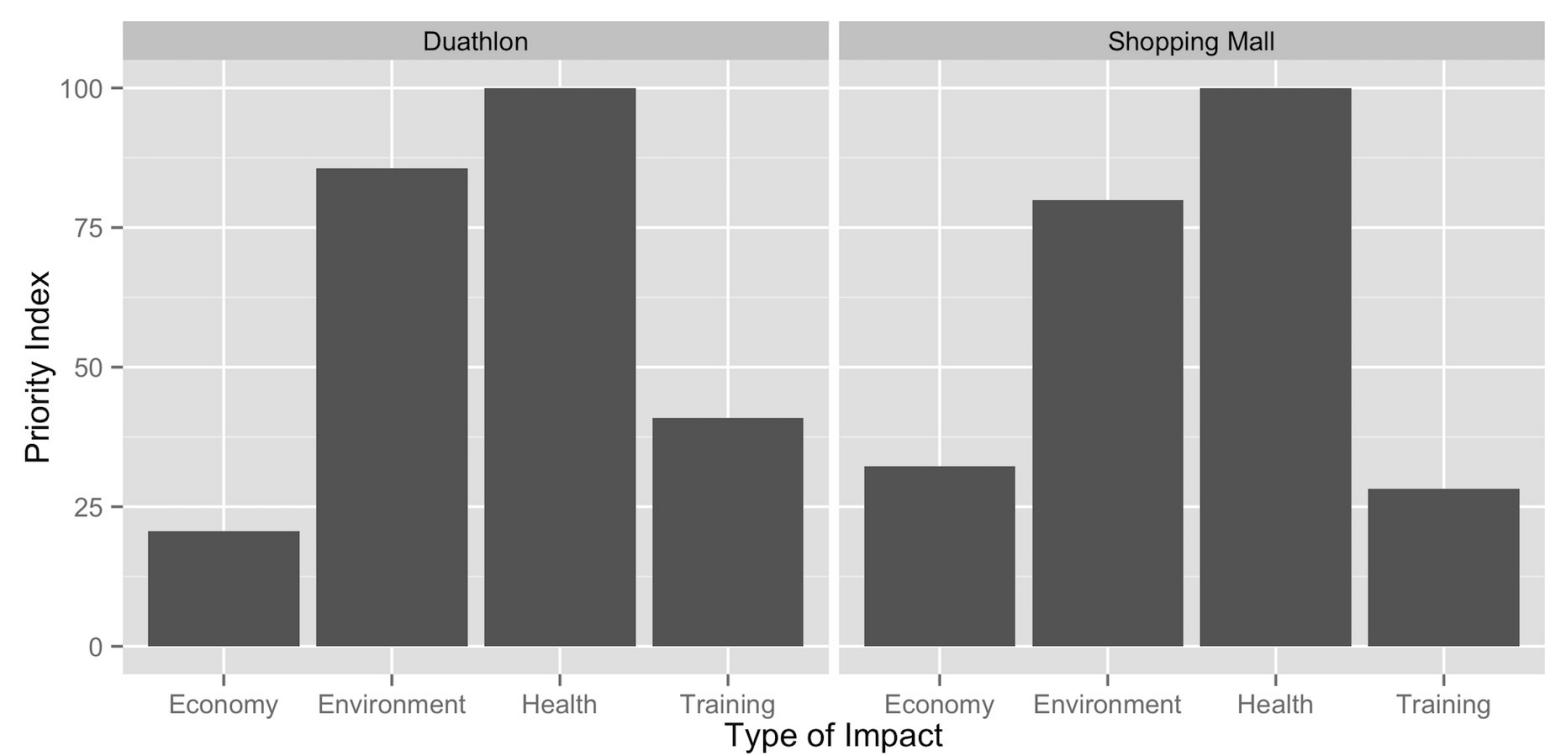
Ranking of impacts. Ranking of concerns about different types of haze impacts (economic, environmental, health, and on sports training).

We asked respondents to rank the importance of four types of impacts of the haze. We normalized their responses using a ‘Relative Priority Index’ (RPI) that was calculated by: (1) for each respondent we gave a score of 3, 2, 1, and 0 to each item in decreasing order of priority; (2) the scores of each item were summed in each population (duathlon vs. mall), and (3) normalized (in each population) by setting the highest priority item to 100 and expressing all further items as a percentage of the highest priority item. Respondents ranked health impacts as the most important (RPI = 100), followed closely by impacts on the environment (RPI = 82.4) and, with much lower priority, the impacts on their sports training (RPI = 33.8) and the economy (RPI = 27.2). This pattern did not change significantly when we compared respondents at the duathlon and the shopping mall (**χ** ^2^ = 12, df = 9, *p*<0.05; Fig 1).

### Current and future perspectives on air quality

**Fig 2:**
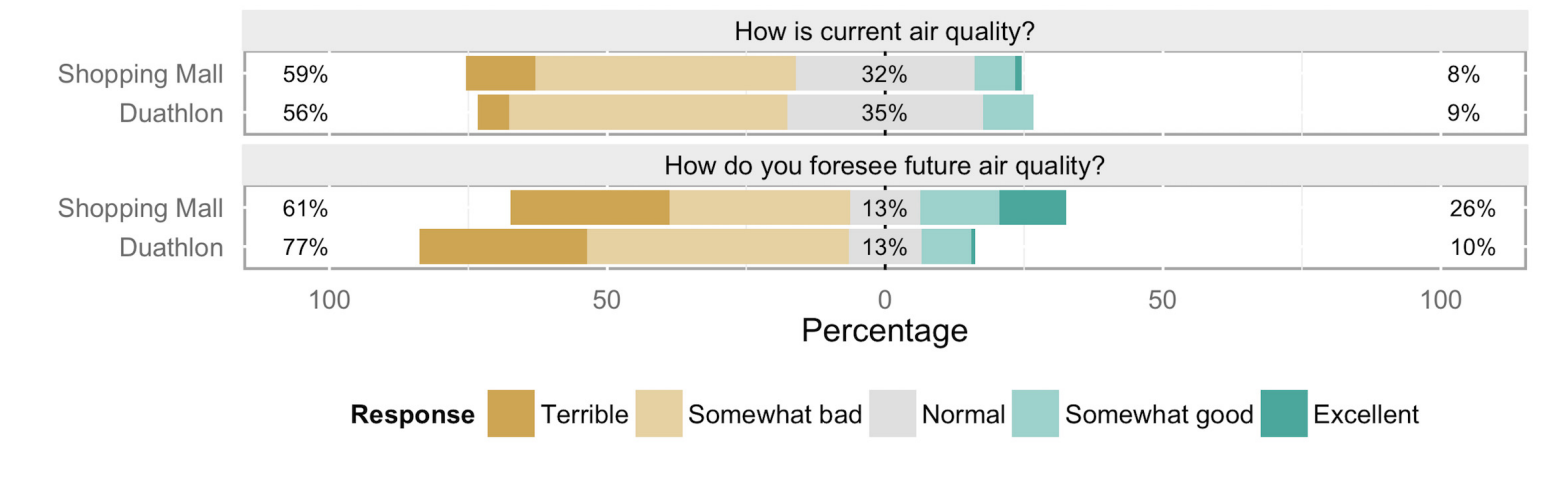
Perception of air quality. Comparison of current and projected perceptions of air quality levels.

The majority of respondents across both populations rated the current air quality as somewhat bad or terrible (59% at the mall, 56% at the duathlon; Fig 2). When asked about future air quality there was a dramatic increase in extremely negative answers at both sites, with many more respondents expecting air quality to be ‘terrible’ in the future. In the shopping mall, there was also a three-fold increment in positive answers (from 8% to 26% of respondents; Fig 2).

### Awareness and concern

#### Awareness and factors affecting it

**Fig 3:**
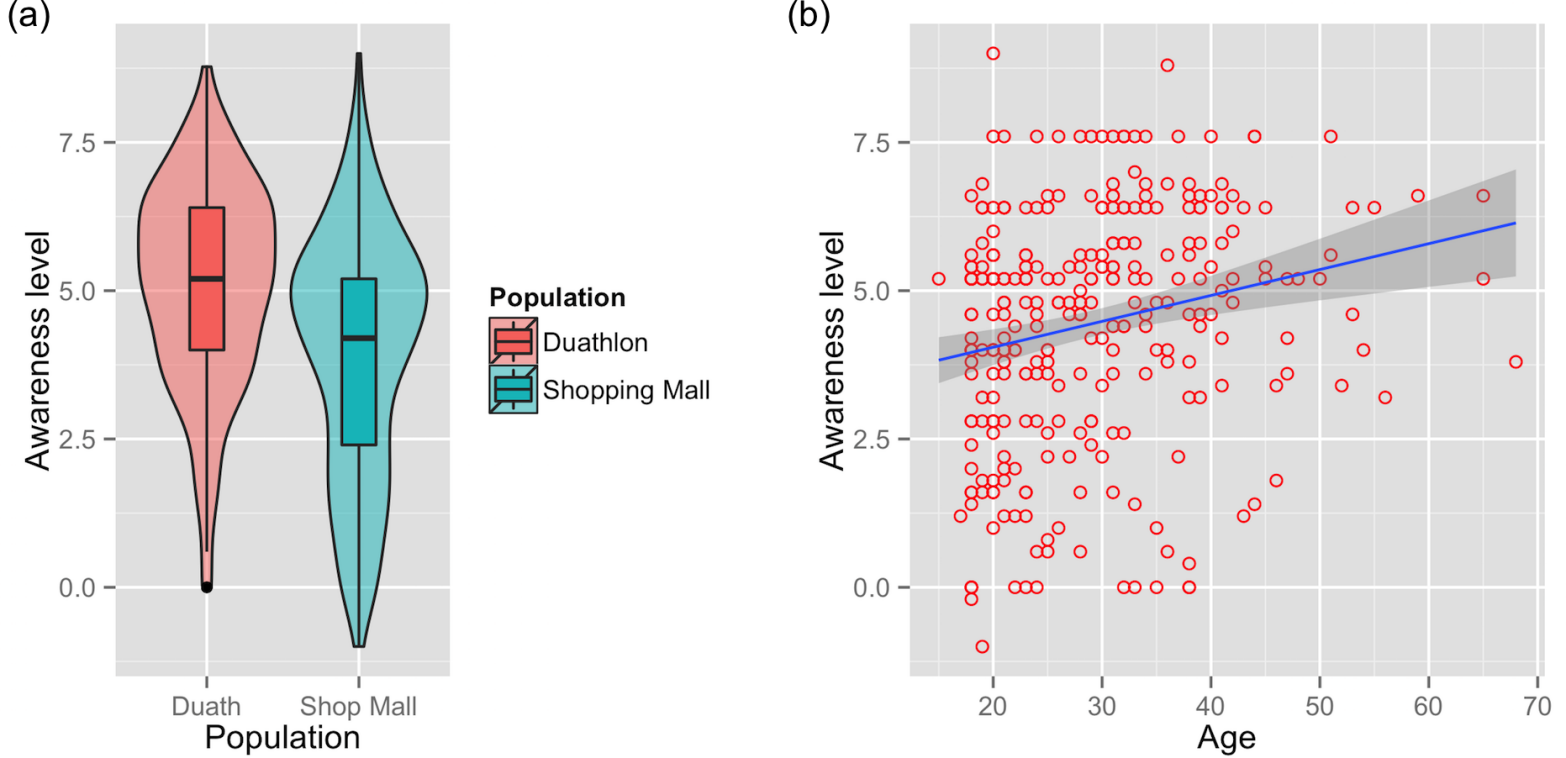
Awareness. Knowledge about the haze across entire sample, by (a) location of survey and (b) age. The shaded area around the regression line in (b) represents a loess smoothed conditional mean.

We defined the mean level of knowledge about the haze as ‘awareness’. The mean (± SD) awareness level of all participants was 4.4 ± 2.0 (range = -1 to 9, due to the penalty applied for incorrect answers), indicating that the questions were posed at an appropriate level of difficulty for the targeted sample. Comparing both populations, the awareness level was higher among duathletes (5.1 ± 1.7 vs. 4.0 ± 2.1; *p*<0.05; Fig 3A and Table 3). Duathletes, who we assume to be more exposed to the negative effects of the haze, were more knowledgeable about the haze than the general public. A number of specific differences in the overall sample disappeared when the subsample of Malaysian nationals at the mall were tested (Table 3). Male respondents had higher levels of awareness than females (4.7 ± 2.0 vs. 4.0 ± 1.9; *p*<0.05), but this gender difference disappeared when testing only the subsample of Malaysian nationals at the shopping mall (4.3 ± 2.1 vs. 3.8 ± 1.9; *p*>0.05). Age also had a significant effect across the whole sample, with older respondents showing higher awareness levels (b ± SE = 0.044 ± 0.011; F_1,298_ = 14.6, *p*<0.05; Fig 3B), but this effect also disappeared when we tested only among Malaysian respondents at the shopping mall (F_1,149_ = 2.3, *p*>0.05). Nationality (Malaysian vs. non-Malaysian) itself had no effect on haze awareness level (5.0 ± 2.1 for Malaysians vs. 4.7 ± 1.9 for non-Malaysians; *p*>0.05).

**Table 3:** Distribution of awareness and concern.

		Awareness	Concern
**Overall mean**		4.4 ± 2.0(range = -1 to 9)	3.9 ± 0.5(range = 2.1 to 4.8)
**Mall respondents**		4.0 ± 2.1	3.8 ± 0.5
**Duathlon respondents**		5.1 ± 1.7	3.9 ± 0.4
	*t*	5.3	2.6
	df	291.7	283.2
	p-value	p = 2.8e-7	p = 0.011
**All respondents practicing sport**		4.6 ± 2.0	3.9 ± 0.5
**All respondents non practicing sport**		3.8 ± 2.0	3.8 ± 0.5
	*t*	3.2	2.6
	df	138.2	129.7
	p-value	p = 0.002	p = .067
**Mall Malaysian respondents practicing sport**		4.1 ± 2.1	3.8 ± 0.5
**Mall Malaysian respondents non practicing sport**		3.7 ± 2.0	3.8 ± 0.5
	*t*	1.3	0.35
	df	167	119.4
	p-value	p = 0.196	p = .73
**Malaysian respondents**		5.0 ± 2.1	3.9 ± 0.5
**Non Malaysian respondents**		4.7 ± 1.9	3.8 ± 0.4
	*t*	-0.7	-1.7
	df	67.6	75.2
	p-value	p = 0.482	p = 0.096
**Male respondents**		4.7 ± 2.0	3.9 ± 0.5
**Female respondents**		4.0 ± 1.9	3.9 ± 0.4
	*t*	-2.9	0.26
	df	280.7	274.2
	p-value	p = 0.004	p = 0.8
**Mall Malaysian male respondents**		4.3 ± 2.1	3.7 ± 0.5
**Mall Malaysian female respondents**		3.8 ± 1.9	3.9 ± 0.5
	*t*	-1.6	1.46
	df	129.2	125.4
	p-value	p = 0.121	p = 0.15
**Income**	< RM2500	4.3 ± 2.1	3.8 ± 0.5
	RM2500-5000	4.4 ± 1.9	3.8 ± 0.4
	RM5000-7500	5.2 ± 1.5	4.0 ± 0.5
	RM7500-10,000	3.9 ± 2.2	3.9 ± 0.6
	> RM10,000	4.7 ± 2.1	3.9 ± 0.4
	*df*	(4, 189)	(4, 189)
	F	1.93	0.55
	p-value	0.11	0.70
**Income among Mall Malaysian respondents**	< RM2500	3.8 ± 2.1	3.8 ± 0.5
	RM2500-5000	4.3 ± 1.9	3.7 ± 0.5
	RM5000-7500	4.7 ± 1.7	3.9 ± 0.4
	RM7500-10,000	3.9 ± 2.3	3.8 ± 0.6
	> RM10,000	4.7 ± 1.0	3.7 ± 0.1
	*df*	(4, 89)	(4, 89)
	F	0.69	0.23
	p-value	0.60	0.92
**Education**	Primary*	2.5 ± 2.4	3.9 ± 0.2
	Secondary	4.5 ± 1.9	3.8 ± 0.5
	Tertiary (graduate)	4.5 ± 1.9	3.9 ± 0.5
	Postgraduate (masters/PhD)	4.3 ± 2.2	3.8 ± 0.5
	*df*	(2, 295)	(2, 292)
	F	0.25	0.81
	p-value	0.78	0.45
**Education among Mall Malaysian respondents**	Primary*	1.1 ± 0.7	3.9 ± 0.2
	Secondary	3.9 ± 1.8	3.8 ± 0.6
	Tertiary (graduate)	4.2 ± 1.9	3.8 ± 0.5
	Postgraduate (masters/PhD)	4.0 ± 2.6	3.7 ± 0.5
	*df*	(2, 144)	(2, 142)
	F	0.44	0.34
	p-value	0.64	0.71
**Having children**	With children	4.5 ± 2.2	3.8 ± 0.5
	Without children	4.4 ± 1.9	3.9 ± 0.5
	*df*	157.9	172.5
	t	0.67	-1.04
	p-value	0.50	0.30
**Having children among Mall Malaysian Respondents**	With children	3.8 ± 2.2	3.7 ± 0.5
	Without children	4.0 ± 1.9	3.8 ± 0.5
	*df*	42.9	42.2
	t	-0.65	-1.54
	p-value	0.52	0.13
**Suffering health condition**	With condition	4.5 ± 2.2	3.9 ± 0.5
	Without condition	4.4 ± 1.9	3.9 ± 0.5
	*df*	121.1	125.3
	t	0.36	0.29
	p-value	0.72	0.77
**Suffering health condition among Mall Malaysian Respondents**	With condition	4.3 ± 1.9	3.8 ± 0.5
	Without condition	3.9 ± 2.1	3.8 ± 0.5
	*df*	102.4	96.4
	t	1.19	0.58
	p-value	0.24	0.57

* respondents with only primary education were excluded from the analyses because of the small sample size (n = 3).

Education level had no significant effect on haze awareness level (we excluded primary education because there were just three respondents in this category), not even when we tested only Malaysians at the shopping mall. Income also had no significant effect on awareness level, neither across the overall sample nor among Malaysians at the shopping mall (Table 3). Education and income were positively correlated (*t* = 3.26, df = 155, *p*<0.05).

Respondents who regularly practice outdoor sport (including duathletes, but also others who stated that they practiced outdoor sports regularly) showed higher levels of awareness than those who do not (4.6 ± 2.0 vs. 3.8 ± 2.0; *p*<0.05). This result indicated *prima facie* that practicing outdoor sport at any level correlates to higher awareness over the phenomenon of the haze. However, when we repeated the analysis including only the subsample of Malaysian respondents interviewed at the shopping mall, there was no significant difference in awareness between those who practice sports (4.1 ± 2.1) and those who do not (3.7 ± 2.0; *p*>0.05). Among respondents who practice sport regularly, we found no effect of the number of weekly training hours, nor its interaction with the population (shopping mall vs. duathlon event), on awareness levels (F_3, 145_ = 0.42, *p*>0.05)

**Fig 4:**
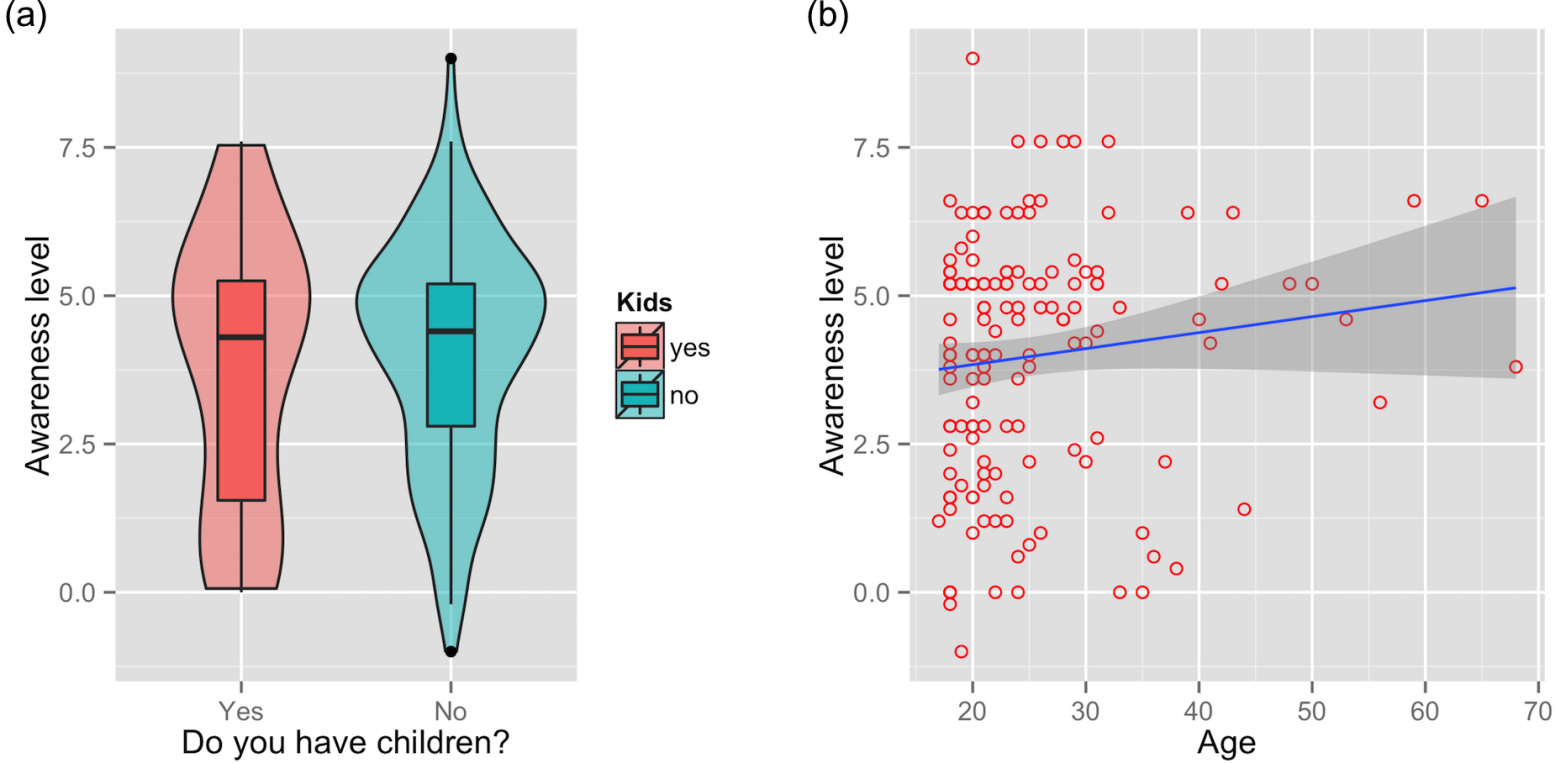
Awareness at shopping mall. Knowledge about the haze amongst Malaysians at a shopping mall, by parenthood and age. The shaded area around the regression line in (b) represents a loess smoothed conditional mean.

The minimum adequate model retained just two variables: population (duathletes vs. people at the shopping mall; *t* = -3.81, df = 297, *p*>0.05; Fig 3A) and age (b = 0.027; *t* = 2.250, df = 297, *p*<0.05; Fig 3B). For Malaysian respondents at the shopping mall, the minimum adequate model also retained just two variables: parenthood (*t* = 2.17, df = 148, *p*<0.05; Fig 4A) and age (b = 0.059; *t* = 2.57, df = 297, *p*<0.05; Fig 4B).

#### Concern and factors affecting it

We defined the mean of all retained scalar attitudinal questions as ‘concern’. The mean (± SD) concern level was 3.9 ± 0.5 (range 2.1–4.8). Concern level was slightly but significantly higher among respondents at the duathlon event than at the shopping mall (3.94 ± 0.4 vs. 3.80 ± 0.5; *p*<0.05; Table 3). In addition, people who regularly practice sports (without necessarily being duathletes) showed a tendency towards greater concern compared to those who do not practice sport regularly (3.89 ± 0.46 vs. 3.77 ± 0.50; *p =* 0.067).

Gender had no effect on concern level, neither on the whole population nor among Malaysians at the shopping mall. Age had a positive effect on concern over the whole population but this effect disappeared when we tested only Malaysians at the shopping mall. Nationality had a marginal effect, with Malaysians showing a tendency towards higher concern than non-Malaysians (3.87 ± 0.48 vs. 3.76 ± 0.44, *p =* 0.096). The level of education (*p*>0.05 for both the whole sample and for just Malaysians at the shopping mall) and income (*p*>0.05 for both the whole sample and for just Malaysians at the shopping mall) had no significant effect on concern levels. See Table 3 for more details on factors affecting concern.

**Fig 5:**
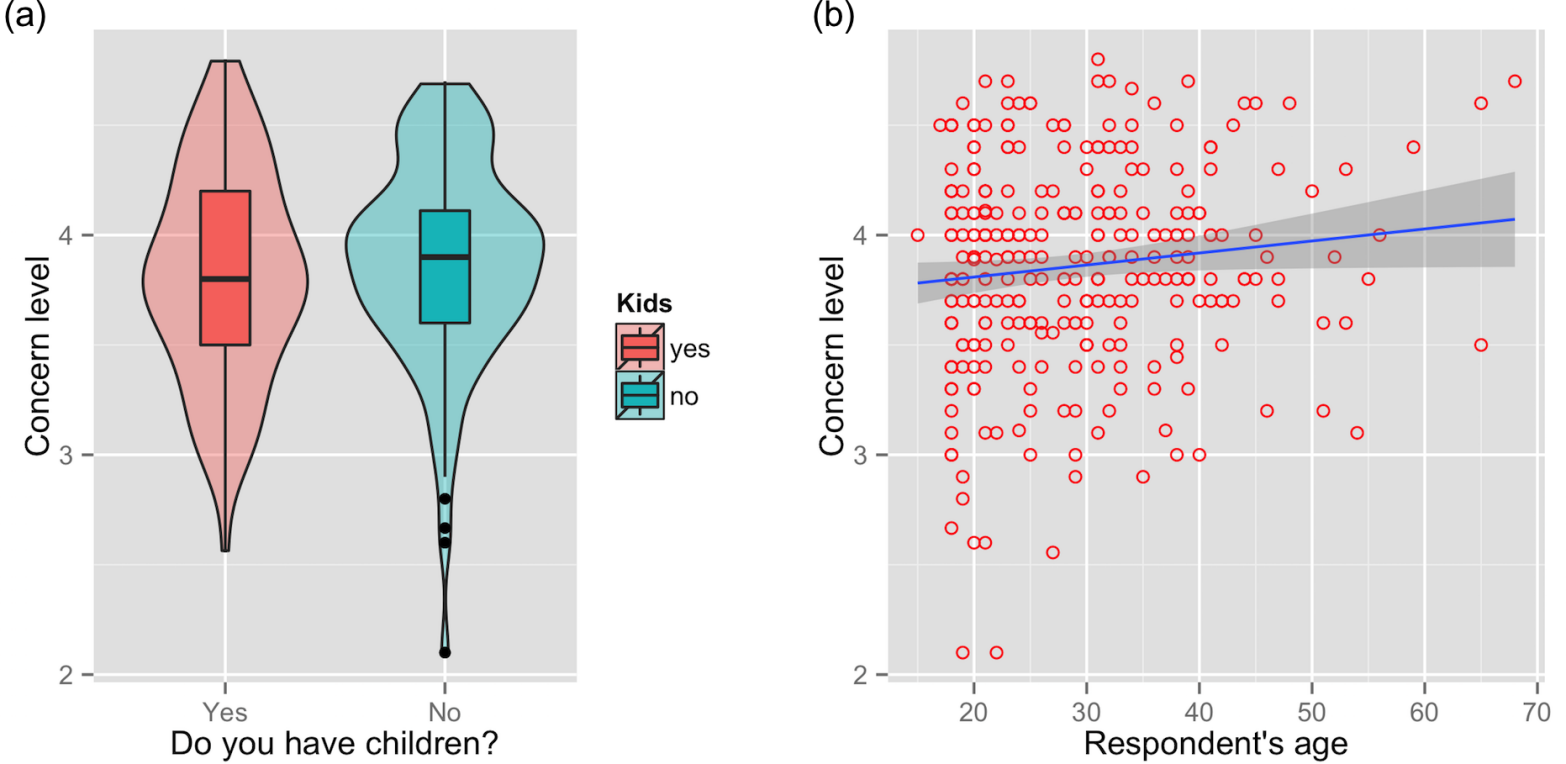
Attitudes towards haze. Level of concern about the haze across the entire sample, by parenthood and age. The shaded area around the regression line in (b) represents a loess smoothed conditional mean.

**Fig 6:**
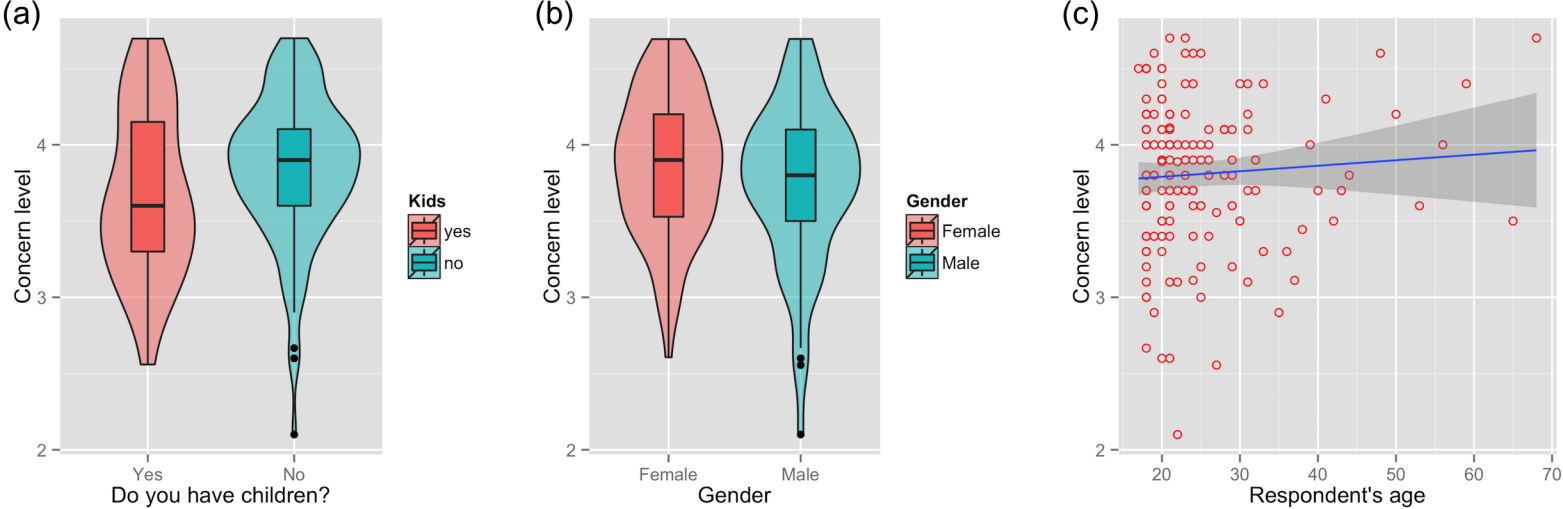
Attitudes towards haze at shopping mall. Level of concern about the haze amongst Malaysians at the shopping mall, by parenthood, gender, and age. The shaded area around the regression line in (c) represents a loess smoothed conditional mean.

**Fig 7:**
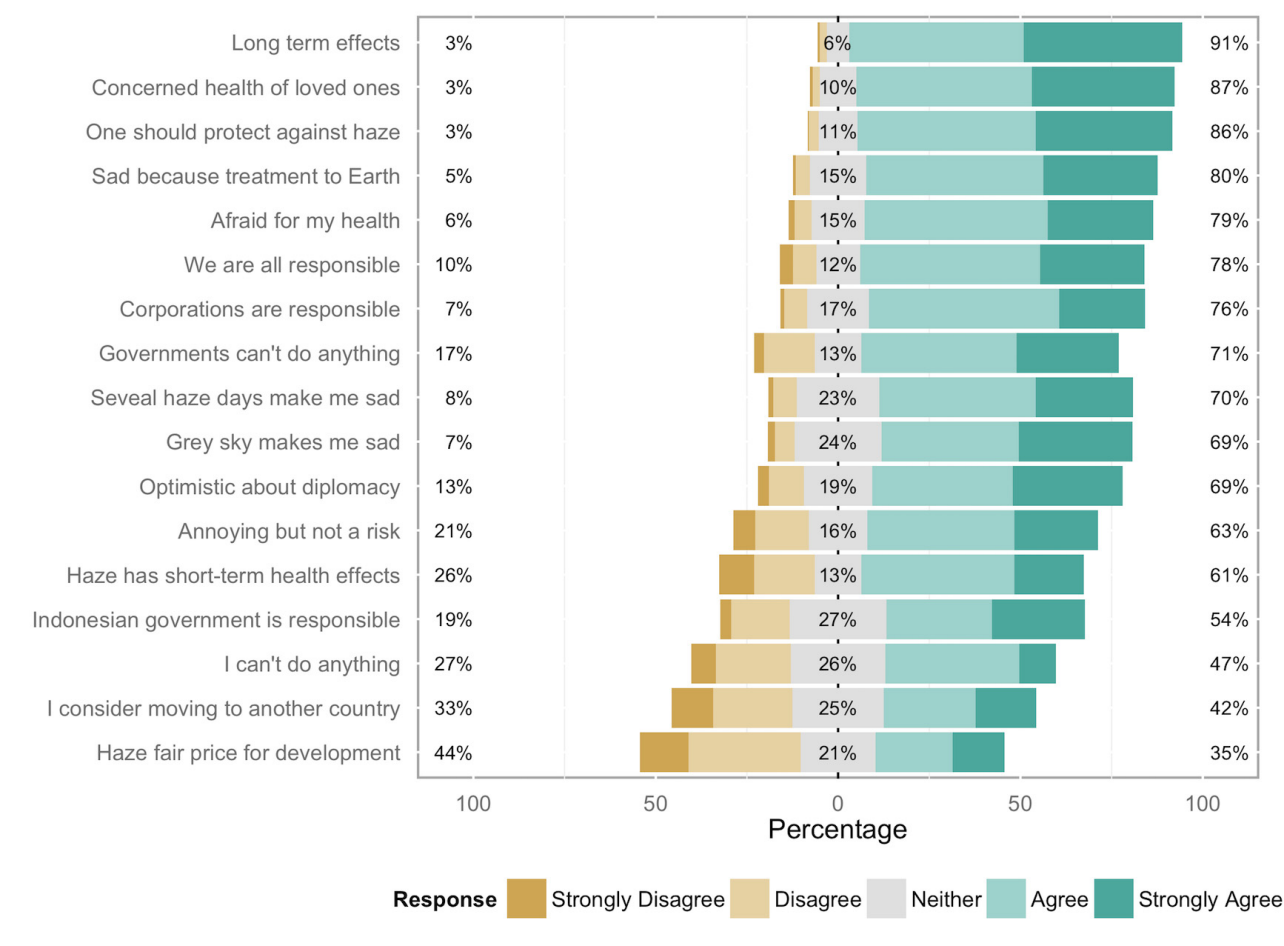
General attitudes towards haze. Raw answers to all attitude questions by the entire sample.

The minimum adequate model for both populations again retained just two variables: parenthood (*t =* 2.90, df = 294, *p*<0.05; Fig 5A) and age (*t =* 3.35, df = 294, *p*<0.05; Fig 5B). The minimum adequate model for Malaysians interviewed at the shopping mall retained three variables: gender (*t =* -1.99, df = 145, *p*<0.05; Fig 6A), parenthood (*t =* 3.43, df = 294, *p*<0.05; Fig 6B), and age (b = 0.015, *t =* 2.92, df = 294, *p*<0.05; Fig 6C). The raw answers to all attitude questions are shown in Fig 7.

#### Awareness and Concern

**Fig 8:**
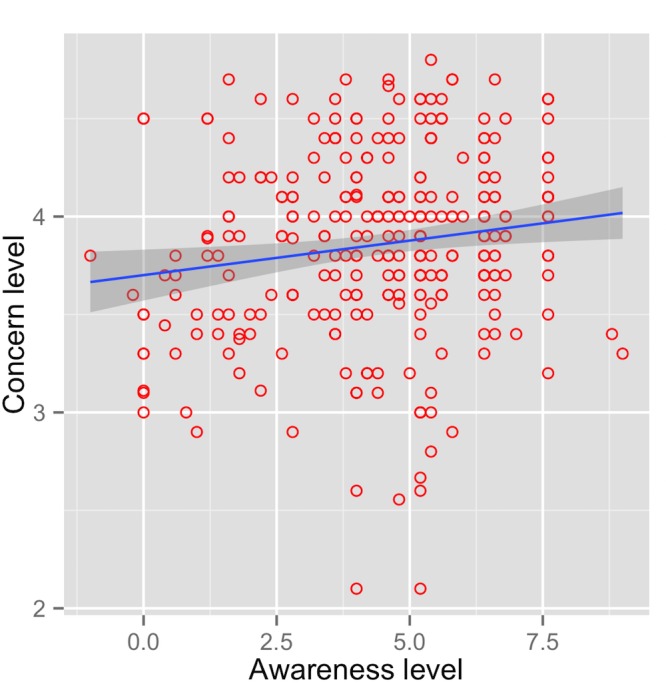
Knowledge and attitudes. Correlation between haze awareness levels and the level of concern about haze effects across the entire sample. The shaded area around the regression line represents a loess smoothed conditional mean.

The level of concern (representing Attitudes) showed a weak positive correlation with awareness (representing Knowledge) (r = 0.15, *t =* 2.6, df = 300, *p*<0.05; Fig 8). Thus hypothesis 1 was accepted; people with greater knowledge about the haze also report greater concern about its effects.

### Behavioural responses to awareness and concern

In relation to Practices, we asked respondents whether they checked the air quality readings (the Malaysian Air Pollution Index (API) or the Singaporean Pollutant Standards Index (PSI)–hereafter API/PSI on a daily basis, and whether they had ever cancelled training sessions due to the severity of the haze. The percentage of people that checked the API/PSI on a daily basis was significantly higher among respondents at the duathlon event (15.9% vs. 4.9%; *p*<0.05; Table 3). Among those who practiced sports regularly, respondents at the duathlon event were more likely to check the API/PSI index before practising sport (40.5% vs. 14.5%; *p*<0.05; Table 3). Against our expectations, people who reported a pollution-sensitive health condition were not more likely to check the API/PSI levels daily than those with no condition (odds ratio 1.2 times; **χ** ^2^ = 0.14, p>0.05).

People who practice outdoor sports (in total, not only duathletes in Port Dickson) who have cancelled outdoor sport sessions due to the haze showed a higher awareness level than those who had not cancelled sessions (5.1 ± 1.7 vs. 4.2 ± 2.2; *t =* 2.58, df = 77.2, *p*<0.05). Similarly, amongst the same group, those who check API/PSI levels before practicing outdoor sport also showed higher awareness levels than those who do not check the indexes (5.1 ± 1.8 vs. 4.4 ± 2.0; *t =* 2.55, df = 117.9, *p*<0.05). With regards to concern, there was a higher level of concern among people who practice outdoor sports and report cancelling outdoor training sessions (3.99 ± 0.43 vs. 3.71 ± 0.44; *t =* 3.29, df = 63.7, *p*<0.05), although there was no effect on concern level of the number of hours of training (F_1,147_ = 0.04, *p*>0.05). Thus, hypothesis 2 was also accepted; greater knowledge and concern over the haze means that people are more likely to engage into protective actions against it, such as cancelling outdoor sports training sessions. However, it was also observed that higher levels of haze concern amongst outdoor sportspeople (*t =* 0.64, df = 96.6, *p*>0.05) did not increase the likelihood of checking the API/PSI daily.

## Discussion

A Knowledge-Attitudes-Practices approach has been adopted here with three hypotheses, all of which have been accepted in the results of the study. In regards to knowledge, the haze crisis of 1997/98 was associated with widespread misperception of the causes of haze, which were primarily regarded as a 'natural disaster' despite the fires being deliberately lit for land-clearing purposes [25]. Worried about negative effects on tourism, the Malaysian government used the Official Secrets Act [26] to stifle media reports about air pollution from 1999 until 2005, which limited public discussion of the problem during this period [27]. This may explain why a large number of people surveyed in the shopping mall lacked even basic awareness of the phenomenon, and also the curious fact that education level was not positively correlated with knowledge about the causes and nature of haze episodes (particularly striking given that some of the questions used to measure knowledge were of a technical and/or factual nature). As hypothesised, the group of duathletes did show a significantly higher level of awareness about the haze in comparison with the shopping mall sample.

Besides participation in the duathlon, the only other significant predictor of haze awareness was age. Younger respondents scored lower in awareness than older respondents. Since age appears to correlate with awareness but not with concern, it is unlikely that this difference is explainable in reference to poorer health amongst the elderly; it is also the case that very few respondents were of such advanced age that one might expect greater respiratory sensitivity, for example. The likely interpretation therefore is that older people are able to recall the very severe haze episode of 1997, which was so extreme as to be declared a national emergency, and was widely reported in the media both locally and internationally prior to the abovementioned imposition of media restrictions by the government.

In regards to attitudes, as could be expected, general attitudes towards the haze are highly negative amongst the overall population. The marked public discomfort with the phenomenon is starkly revealed by the 42% of respondents who reported that they had considered leaving the country due to its effects (Fig 7). A vast majority of the people surveyed are worried about the health of themselves and their loved ones (79% and 87% respectively) and the negative effects on the natural environment (80%), and most people surveyed reported sadness in response to haze conditions (70%) (Fig 7). Our results show that concern over the haze is overwhelmingly due to its effects on human health and the health of the natural environment, with economic effects rated last on a scale of negative impacts. This contrasts with the reported attitude of the Malaysian government, which justified the withholding of information about the severity of haze episodes between 1999 and 2005 on the grounds that publication would potentially affect tourism and thus have a negative economic impact [28,29]. Nonetheless, 35% of respondents were willing to accept that haze events are ‘a fair price to pay for economic development’ (Fig 7), so the notion that Malaysia’s status as a rapidly developing nation demands some health and environmental sacrifices resonated with a reasonably large minority, who may have adopted the idea of a necessary connection between economic growth and damage to the environment and public health.

As hypothesised, of the two populations studied–participants in a duathlon and people wandering through a popular shopping mall–duathletes showed significantly greater levels of concern about the haze in comparison to non-duathletes. This is consistent with our findings that point to health effects as being the major concern of Malaysian people in regards to air pollution. The hypothesised correlation between level of awareness (knowledge) and level of concern (attitudes) was also evident, although weak. It could be that those who are more concerned about the effects of the haze are somewhat more likely to seek information about its features and causes. Duathletes in Port Dickson showed much higher levels of concern about the haze phenomenon than respondents in the shopping mall. Amongst non-duathletes, there was no difference in concern between those who reported participating in outdoor sporting activities and those who did not, suggesting that greater concern is found amongst keen athletes rather than merely ‘sporty’ people.

In regards to practices, concern over the effects of air pollution on their health regularly convinced the duathletes to cancel training sessions when high levels of pollution were in effect, thus the hypothesised link between concern and taking protective action appears to be in place. Amongst duathletes, there was also a positive correlation between their level of knowledge about the haze and the taking of protective behaviours in the form of cancelling training sessions and daily monitoring of API/PSI levels. A large majority (73.5%) of all respondents who engage in outdoor sporting activities report having cancelled outdoor training sessions because of the haze; this is especially evident amongst duathletes. Among the same group (all sportspeople), 30.5% report also checking the API/PSI before participating in outdoor sport, but again this is much more evident among duathletes. The daily checking of API/PSI levels was prompted by higher levels of awareness, not by higher levels of concern. It therefore appears that outdoor sportspeople who have greater levels of knowledge about the haze take some preventive actions (cancelling outdoor sport sessions) based on quantitative evidence (checking the API/PSI), whereas outdoor sportspeople who have greater levels of concern about the haze (but know relatively less about its causes and effects) take some preventive actions without seeking objective measures.

Compared to general air pollution, transboundary haze is more directly traceable to its source; political solutions remain elusive, nonetheless, due to patronage networks, the dispersion of blame, and other impediments to effective regulation [4, 30–31]. The present study reflects this in the generally negative expectations that were reported as to future air quality; there was a notable shift in expectations towards extremely bad air quality, indicating pessimism with regards to the likelihood of solving or even mitigating the problem of transboundary haze. Despite their general pessimism, 69% of the sample reported optimism about a diplomatic solution to the haze crisis. On this point it is worth noting that at the time of conducting the surveys, a major diplomatic breakthrough had just occurred. In September 2014, Indonesia (on the territory of which most of the open burning that is the main cause of the haze has taken place) agreed to become the last ASEAN nation to ratify the ASEAN Agreement on Transboundary Haze Pollution [32], 12 years after the agreement was established [33]. It is likely that this event was fresh in the minds of many respondents, who therefore felt a diplomatic solution was imminent.

Some limitations were identified in this study. Firstly, in relation to practices the survey only asked participants about cancelling training sessions and checking API/PSI levels. It would be useful to look at a wider range of behaviours in response to haze conditions, such as wearing face masks, staying indoors, etc. Secondly, the element of the survey intended to measure psychological wellbeing was found to be unreliable. While it was not among the main purposes of this study, an effective measure of wellbeing and stress levels would be of great interest in identifying the effects of haze conditions and how any impairment to personal wellbeing translates into adaptive behaviours. Thirdly, the two locations in which the surveys were conducted varied greatly across a number of demographic variables. As discussed above, the impact of this variance on the results was mitigated by strategies adopted in analysis; nonetheless a more homogenous sample may have revealed clearer and more immediate differences between sportspeople and the general population. Additionally, further studies could be done with other categories of particularly vulnerable people, such as children, the elderly, and pregnant women to better understand what factors determine people’s awareness, attitudes, and practices in relation to the transboundary haze.

## Conclusions

In this study we examined levels of awareness, concern, and protective behaviour amongst people in Malaysia in regards to the transboundary air pollution that has affected large swathes of Southeast Asia on a seasonal basis over the past three decades. Duathletes, for whom the haze phenomenon represents a greater risk to health and a greater impediment to lifestyle, were more knowledgeable about transboundary haze when compared to a general sample of shopping mall visitors. Many of the latter had very little awareness of the causes and effects of haze, and the level of education was an insignificant factor. Amongst the entire sample, the overwhelming concern of Malaysian residents in regards to the haze is its effects on their health and the health of their loved ones. While over a third of respondents were willing to accept haze-related air pollution as one of the unavoidable facts of life in a developing economy, public attitudes towards the phenomenon are strongly negative, with an even higher percentage of respondents stating that they had considered leaving the country altogether because of haze events. Sportspeople who know more about the haze are more likely to demonstrate concerned attitudes towards it, and also more likely to engage in practices that help to avoid the worst effects, such as cancelling training sessions. Providing accurate and timely information, both through the media and the education system, is therefore likely to lessen the human impact of haze events in Malaysia, especially on those most vulnerable to its effects.

## Supporting Information

S1 File Survey (English). (PDF)

S2 File Survey (Bahasa Melayu). (PDF)
