# Supplementary material for: The Link between Knowledge, Attitudes and Practices in Relation to Atmospheric Haze Pollution in Peninsular Malaysia
Source: PLoS One. 2015 Dec 8;10(12):e0143655. doi: 10.1371/journal.pone.0143655 (PMC4672926; doi:10.1371/journal.pone.0143655)
Supplement: S1 File — (PDF) [file pone.0143655.s001.pdf]

Study on Awareness and Attitudes Towards Haze

LL	DD	PP	NN

Dear Participant,

This is a survey regarding awareness of and attitudes towards the haze in Malaysia and Singapore, and how the haze influences people's wellbeing. It is implemented by a team of researchers from the University of Nottingham Malaysia Campus. Your participation is voluntary and you may discontinue at any time. We assure you of the following:

- Responses are anonymous, and the individual study results will be confidential. Data will not be traceable to you and it will not be shared with anyone besides the researchers.
- There are no known recognized risks to your participation in this study.

Thank you for your time and desire to help in this research. If you have any questions, or if you want the study's final report, please contact the leading researcher Laura De Pretto at Laura.DePretto@nottingham.edu.my.

A) BACKGROUND INFORMATION

How old are you?

______ yrs.

What is your gender? (Please circle)

1. Female
2. Male

What is your ethnicity?

1. Malay
2. Chinese
3. Indian
4. Other (please specify: ____________________)

What is the highest educational level you have completed?

1. Primary
2. Secondary
3. Tertiary (university / college)
4. Postgraduate (master / PhD)

What is your occupation? __________________________

What is your household income? (in RM per month; RM1000 ~ SGD390)

1. < 2,500 (< SGD1000)
2. 2,501 – 5,000 (SGD1000-2000)
3. 5,000 – 7,500 (SGD2000-3000)
4. 7,501 – 10,000 (SGD3000-4000)
5. > 10,000 (> SGD4000)
6. I prefer not to answer

Do you have children? (Please circle)

1. Yes
2. No

Do you have any health condition that makes you more sensitive to poor air quality than other people?

1. Yes
2. No

What is your nationality?

____

What is your country of residence?

____

Do you regularly practice outdoor sports?

1. Yes
2. No

Are you taking part in Port Dickson's International Duathlon?

1. Yes
2. No

If "yes", what distance?<br><br>1. Full<br>2. Sprint	In how many duathlon / triathlon races (any distance) have you participated before this one?<br><br>______ races
On average, how many hours per week have you trained outdoors in the past three months?<br><br>______ hours per week.	Have you cancelled any outdoor training sessions because of the haze?<br><br>1. Yes<br>2. No
Do you check the API / PSI daily?<br><br>1. Yes<br>2. No	Do you check the API / PSI before practicing outdoor sport or exercise?<br><br>1. Yes<br>2. No
ONLY FOR NON MALAYSIANS AND NON SINGAPOREANS:	How long have you lived in Malaysia/Singapore for? ______________<br><br>How long do you think you will stay in Malaysia/Singapore? ________

B) HAZE AWARENESS

Below are a number of statements about the haze in Malaysia and Singapore. For each item, please circle whether you believe the statement is more likely to be TRUE or FALSE. If you *really* have no idea, then you can circle NA.

The Malaysian Department of Environment publishes an official Air Pollution Index (API) which is updated every hour. It is similar to Singapore's Pollutant Standards Index (PSI). A API / PSI value of 151 is considered 'unhealthy'.	TRUE	FALSE	NA
During the period Jan-Sep 2014, the API in Kuala Lumpur was rated as 'unhealthy' on more than 20% of days.	TRUE	FALSE	NA
The haze affecting peninsular Malaysia over the past two years was most often caused by fires in Thailand.	TRUE	FALSE	NA
Severe haze episodes over the past few years were caused by the burning of underground peatland soil.	TRUE	FALSE	NA
June 2013 was the period in which the highest levels of haze API / PSI were recorded in Singapore and Malaysia.	TRUE	FALSE	NA
According to the WWF, the estimated economic cost of the large-scale fires in Indonesia during 1997/98 was USD100 million.	TRUE	FALSE	NA
It is estimated that between 15-40% of global CO_2 emissions in 1997 were due to the fires in Indonesia.	TRUE	FALSE	NA
Malaysian API standards are more stringent than World Health Organisation guidelines.	TRUE	FALSE	NA
During moderate exercise, a person intakes up to 500% more pollutant matter due to enhanced breathing.	TRUE	FALSE	NA
Both large corporations and small landholders responsible for the fires that cause the haze in Sumatra.	TRUE	FALSE	NA

If you would like to know the correct answers, please email Laura.DePretto@nottingham.edu.my

C) HAZE ATTITUDES

Below are statements that describe how you may think and feel about the haze. Please use the following scale to indicate your level of agreement or disagreement with each statement.

1	2	3	4	5
Strongly Disagree	Disagree	Neither Agree nor Disagree	Agree	Strongly Agree

With regards to the haze:	Strongly Disagree	Disagree	Neither agree nor disagree	Agree	Strongly Agree
1. Nothing can be done by the Malaysian / Singaporean governments to improve the situation.	1	2	3	4	5
2. I am afraid for my health.	1	2	3	4	5
3. Looking at the grey sky makes me sad.	1	2	3	4	5
4. Corporations are responsible for the degradation of air quality.	1	2	3	4	5
5. One should take protective actions against the haze (eg wearing masks, staying indoors).	1	2	3	4	5
6. Several consecutive days of haze makes me depressed.	1	2	3	4	5
7. Nothing can be done from my part to improve the situation.	1	2	3	4	5
8. Haze has short-term health effects on the respiratory system.	1	2	3	4	5
9. I sometimes consider moving to a different country with better air quality.	1	2	3	4	5
10. The Indonesian government is responsible for the degradation of air quality.	1	2	3	4	5
11. We are all equally responsible for the degradation of air quality.	1	2	3	4	5
12. I am afraid for the health of my loved ones.	1	2	3	4	5
13. I am annoyed by the haze, but I don't think it represents a risk for health.	1	2	3	4	5
14. Seeing the haze makes me feel sad because I think of how badly we are treating our earth.	1	2	3	4	5
15. Haze has long-term health effects on the lungs and heart.	1	2	3	4	5
16. The haze is a fair price to pay for economic development.	1	2	3	4	5
17. I am optimistic that diplomacy between countries can address the haze problem.	1	2	3	4	5

Overall, how would you rate the air quality in Malaysia and Singapore? (Circle one answer)

1. Terrible
2. Somewhat bad
3. Normal
4. Somewhat good
5. Excellent

How do you foresee air quality in Malaysia and Singapore in 25 years from now? (Circle one answer)

1. Much worse
2. Somewhat worse
3. Same as now
4. Somewhat better
5. Much better

Please rank the following from 1 to 4 based on what concerns you most in relation to the haze. (Number every box)

☐ A. Economic impact

☐ B. Environmental impact

☐ C. General health impact

☐ D. Impact on my training/exercise

What do you think should be done to improve the haze situation?

- ______________________________
- ______________________________
- ______________________________

D) WELL-BEING

Please think about what you have been doing and experiencing <u>during the past three months</u>. Then report how much you experienced each of the following feelings, using the scale below. Circle one number for each item.

1	2	3	4	5
Very Rarely or Never	Rarely	Sometimes	Often	Very Often or Always

<u>During the past three months, I have been feeling:</u>	Very Rarely or Never	Rarely	Sometimes	Often	Very Often or Always
1. Positive	1	2	3	4	5
2. Bad	1	2	3	4	5
3. Pleasant	1	2	3	4	5
4. Happy	1	2	3	4	5
5. Afraid	1	2	3	4	5
6. Contented	1	2	3	4	5
