# Supplementary material for: The Link between Knowledge, Attitudes and Practices in Relation to Atmospheric Haze Pollution in Peninsular Malaysia
Source: PLoS One. 2015 Dec 8;10(12):e0143655. doi: 10.1371/journal.pone.0143655 (PMC4672926; doi:10.1371/journal.pone.0143655)
Supplement: S2 File — (PDF) [file pone.0143655.s002.pdf]

Kajian Tentang Sikap dan Kesedaran Terhadap Jerebu

LL	DD	PP	NN

Kepada responden,
Borang soal selidik ini adalah mengenai sikap dan kesedaran terhadap jerebu yang berlaku di Malaysia dan Singapura, dan bagaimana jerebu mempengaruhi kesejahteraaan masyarakat. Kajian ini dikendalikan oleh sekumpulan penyelidik dari University of Nottingham Malaysia Campus. Penyertaan anda adalah sukarela dan anda berhak untuk menarik diri pada bila-bila masa sahaja. Kami akan memastikan:

- Penyertaan anda adalah tertutup dan sebarang keputusan kajian individu akan dirahsiakan. Data tidak akan didedahkan kepada anda dan tidak akan dikongsi bersama sesiapa melainkan penyelidik.
- Tiada sebarang risiko dalam menyertai kajian soal selidik ini.

Terima kasih kerana meluangkan masa untuk membantu kami dalam kajian ini. Jika anda mempunyai sebarang soalan, atau jika anda ingin mengetahui laporan hasil kajian ini, sila hubungi ketua penyelidik kami Laura De Pretto at Laura.DePretto@nottingham.edu.my.

A) Info Latar Belakang

Berapakah usia anda?<br><br>______ tahun	Apakah jantina anda?<br>(sila bulatkan)<br><br>1. Perempuan<br>2. Lelaki	Apakah bangsa anda?<br><br>1. Melayu<br>2. Cina<br>3. India<br>4. Lain-lain (sila nyatakan: ____________________________)

Apakah tahap pencapaian pendidikan anda yang tertinggi?

1. Rendah
2. Menengah
3. Pengajian tinggi (universiti / kolej)
4. Pascasarjana (master / PhD)

Apakah pekerjaan anda?____

____

Berapakah pendapatan bulanan anda? (dalam RM setiap bulan RM1000 ~ SGD390)

1. < 2,500 (< SGD1000)
2. 2,501 – 5,000 (SGD1000-2000)
3. 5,000 – 7,500 (SGD2000-3000)
4. 7,501 – 10,000 (SGD3000-4000)
5. > 10,000 (> SGD4000)
6. Saya memilih untuk tidak menjawab soalan ini

Adakah anda mempunyai anak? (Sila bulatkan)<br><br>1. Ya<br>2. Tidak	Adakah anda mempunyai masalah kesihatan yang menyebabkan anda lebih sensitive kepada kualiti udara berbanding orang lain?<br><br>1. Ya<br>2. Tidak
Apakah kewarganegaraan anda?<br><br>_________________________________	Apakah negara kediaman anda?<br><br>__________________________________
Adakah anda kerap melakukan aktiviti luar?<br><br>1. Ya<br>2. Tidak	Adakah anda mengambil bahagian dalam pertandingan Duathlon peringkat antarabangsa di Port Dickson?<br>1. Ya<br>2. Tidak

Jika ya, apakah jaraknya ?<br><br>1. Penuh<br>2. Pecut	Berapa banyak pertandingan duathlon/triathlon pernah anda sertai sebelum ini?<br><br>______ acara
Secara purata, berapa jam kah anda telah melakukan aktiviti luar sejak tiga bulan lepas?<br><br>______ jam setiap minggu.	Adakah anda membatalkan sebarang sesi aktiviti luar disebabkan jerebu?<br><br>1. Ya<br>2. Tidak
Adakah anda memeriksa API/PSI setiap hari?<br><br>1. Ya<br>2. Tidak	Adakah anda memeriksa API/PSI sebelum memulakan aktiviti sukan luar atau latihan?<br><br>1. Ya<br>2. Tidak
HANYA UNTUK BUKAN WARGANEGARA MALAYSIA DAN BUKAN WARGANEGARA SINGAPURA	
Berapa lamakah anda telah menetap di Malaysia atau Singapura? ____________	
Berapa lama lagikah anda akan menetap ? ____________	

Soalan hanya untuk mereka yang berada di Port Dickson, bukan di Midvalley

B) HAZE AWARENESS

Berikut adalah beberapa soalan berkaitan jerebu di Malaysia dan Singapura. Sila bulatkan satu jawapan yang anda rasa betul.

Jabatan Alam Sekitar Malaysia mengeluarkan Indeks Pencemaran Udara (IPU) setiap jam. Singapura menggunakan "Pollutant Standard Index(PSI).<br>IPU/PSI bernilai 151 bermaksud "tidak sihat".	BENAR	SALAH	NA
Dalam tempoh Jan-Sep 2014, IPU di Kuala Lumpur adalah pada tahap "tidak sihat" lebih dari 20% masa.	BENAR	SALAH	NA
The haze affecting peninsular Malaysia over the past two years was most often caused by fires in Thailand.	BENAR	SALAH	NA
Keadaan jerebu teruk kebelakangan ini disebabkan oleh pembakaran tanah gambut (bahagian bawah tanah)	BENAR	SALAH	NA
Bacaan IPU/PSI tertinggi dicatatkan di Malaysia dan Singapura adalah pada bulan Jun 2013	BENAR	SALAH	NA
Berdasarkan data WWF, anggaran kos kerosakan ekonomi disebabkan oleh kebakaran besar-besaran di Indonesia pada tahun 1997/1998 adalah USD100 juta.	BENAR	SALAH	NA
Dianggarkan peratus pelepasan gas karbon dioksida CO2 dunia pada tahun 1997 disebabkan oleh kebakaran di Indonesia adalah antara 15-40%	BENAR	SALAH	NA
Tahap Standard IPU Malaysia adalah lebih ketat berbanding garis panduan Organisasi Kesihatan Dunia (WHO).	BENAR	SALAH	NA
During moderate exercise, a person intakes up to 500% more pollutant matter due to enhanced breathing.	BENAR	SALAH	NA

Syarikat besar dan pemilik tanah kecilan adalah bertanggungjawab untuk kebakaran yang menyebabkan jerebu di Sumatra.	BENAR	SALAH	NA

Sekiranya anda berminat untuk mengetahui jawapan kepada soalan di atas, sila e-mel Laura.DePretto@nottingham.edu.my

C) SIKAP ANDA TERHADAP JEREBU

Berikut adalah penyataan yang menghuraikan tentang perasaan dan pendapat anda tentang gejala jerebu. Sila gunakan skala tersebut untuk menyatakan sama ada anda setuju ataupun tidak mengenai setiap penyataan.

1	2	3	4	5
Sangat tidak bersetuju	Tidak bersetuju	Sama ada setuju ataupun tidak	Setuju	Sangat bersetuju

Berkenaan dengan jerebu :	Sangat tidak bersetuju	Tidak bersetuju	Sama ada setuju ataupun tidak	Setuju	Sangat bersetuju
1. Tiada apa yang boleh dilakukan oleh kerajaan Malaysia/ Singapura bagi mengatasi masalah ini.	1	2	3	4	5
2. Saya sangat bimbang dengan kesihatan diri sendiri.	1	2	3	4	5
3. Memandang ke langit yang kelabu membuatkan saya berasa sedih.	1	2	3	4	5
4. Syarikat besar bertanggungjawab untuk penurunan kualiti udara.	1	2	3	4	5
5. Setiap orang harus mengambil langkah berjaga-jaga terhadap isu jerebu (contoh: memakai topeng muka, berada di dalam rumah)	1	2	3	4	5
6. Keadaan jerebu yang berterusan membuatkan saya berasa tertekan.	1	2	3	4	5
7. Tiada apa yang boleh saya lakukan untuk mengatasi masalah ini.	1	2	3	4	5
8. Jerebu mempunyai kesan kesihatan jangka pendek terhadap sistem respiratori.	1	2	3	4	5
9. Kadangkala saya terfikir untuk berpindah ke negara yang mempunyai kualiti udara yang lebih baik.	1	2	3	4	5
10. Kerajaaan Indonesia bertanggungjawab terhadap penurunan kualiti udara.	1	2	3	4	5
11. Kita semua bertanggungjawab terhadap penurunan kualiti udara.	1	2	3	4	5
12. Saya bimbang akan kesihatan orang yang tersayang.	1	2	3	4	5
13. Saya berasa kesal dengan jerebu, tetapi saya berpendapat ianya tidak berisiko untuk kesihatan.	1	2	3	4	5
14. Jerebu membuatkan saya berasa sedih kerana saya terfikir betapa teruknya kita memelihara bumi ini.	1	2	3	4	5

15. Jerebu mempumyai kesan kesihatan jangka panjang terhadap jantung dan paru-paru.	1	2	3	4	5
16. Jerebu adalah harga yang layak dibayar terhadap pembangunan ekonomi masa kini.	1	2	3	4	5
17. Saya yakin bahawa kerjasama diplomatik antara Negara boleh mengatasi masalah jerebu.	1	2	3	4	5

Secara keseluruhan, bagaimana anda menilai kualiti udara di Malaysia dan Singapura?

1. Sangat teruk
2. Agak teruk
3. Normal
4. Agak baik
5. Cemerlang

Bagaimana anda meramalkan kualiti udara di Malaysia dan Singgapura 25 tahun akan datang?

1. Lebih teruk
2. Agak teruk
3. Sama seperti sekarang
4. Agak baik
5. Lebih baik

Sila kategorikan antara yang berikut bermula dari 1 hingga 4 berdasarkan keprihatinan anda terhadap isu jerebu (nombor di setiap kotak):

☐ A. Impak terhadap ekonomi
☐ B. Impak terhadap alam sekitar
☐ C. Impak terhadap kesihatan
☐ D. Impak terhadap latihan dan sukan

Apakah langkah-langkah yang harus dilakukan untuk mengatasi masalah jerebu?

- ______________________________
- ______________________________
- ______________________________

D) KESEJAHTERAAN

Sila fikirkan apakah yang telah anda lakukan dan alami semasa tiga bulan lepas. Kemudiaan, nyatakan seberapa banyak perasaan di bawah yang telah anda alami dengan menggunakan skala tersebut.

1	2	3	4	5
Sangat jarang atau tidak pernah	Sangat jarang	Kadangkala	Seringkali	Sangat kerap dan selalu

Semasa tiga bulan lepas, saya telah mengalami perasaan:	Sangat jarang atau tidak pernah	Sangat jarang	Kadangkala	Seringkali	Sangat kerap dan selalu
1. Positif	1	2	3	4	5
2. Teruk	1	2	3	4	5
3. Seronok	1	2	3	4	5
4. Gembira	1	2	3	4	5
5. Takut	1	2	3	4	5
6. Memuaskan	1	2	3	4	5
